# MFG-E8 Knockout Aggravated Nonalcoholic Steatohepatitis by Promoting the Activation of TLR4/NF-*κ*B Signaling in Mice

**DOI:** 10.1155/2022/5791915

**Published:** 2022-06-20

**Authors:** Jun Hu, Hui Du, Yinglin Yuan, Peipei Guo, Junxia Yang, Xinru Yin, Jin Liu, Shengwang Wu, Jingyuan Wan, Xia Gong

**Affiliations:** ^1^Department of Pharmacology, Chongqing Medical University, China; ^2^Clinical Immunology Translational Medicine Key Laboratory of Sichuan Province, Sichuan Provincial People's Hospital, University of Electronic Science and Technology of China, Chengdu, China; ^3^Department of Clinical Laboratory, Xinqiao Hospital, Army Medical University, Chongqing, China; ^4^Department of Gastroenterology, Institute of Surgery Research, Daping Hospital, Army Medical University, Chongqing, China; ^5^Department of Pharmacy, Chongqing Ninth People's Hospital, Chongqing, China; ^6^Department of Anatomy, Chongqing Medical University, Chongqing, China; ^7^Department of Hematology, Xinqiao Hospital, Army Medical University, Chongqing, China

## Abstract

Nonalcoholic steatohepatitis (NASH) is the common liver disease characterized by hepatic steatosis, inflammation, and fibrosis; there are no approved drugs to treat this disease because of incomplete understanding of pathophysiological mechanisms of NASH. Milk fat globule-epidermal growth factor-factor 8 (MFG-E8), a multifunctional glycoprotein, has shown anti-inflammation and antifibrosis. Here, MFG-E8 was shown to play a key role in NASH progression. Using methionine and choline deficient (MCD) diet-fed mice, we found MFG-E8 knockout exacerbated hepatic damage and steatosis as indicated by increased plasma transaminases activities and hepatic histopathologic change, higher hepatic triglycerides (TGs), and lipid accumulation. Moreover, liver fibrosis and inflammation elicited by MCD were aggravated in MFG-E8 knockout mice. Mechanistically, MFG-E8 knockout facilitated activation of hepatic toll-like receptor 4 (TLR4)/nuclear factor kappa B (NF-*κ*B) signaling pathway in MCD-fed mice. *In vitro* experiment, the TLR4 specific antagonist TAK-242 rescued palmitic acid- (PA-) primed lipid formation and inflammation in MFG-E8 knockout primary murine hepatocytes. These findings indicated that MFG-E8 is involved in the progression of NASH and the possible mechanism by which MFG-E8 knockout exacerbated NASH in mice is associated with activation of the TLR4/NF-*κ*B signaling pathway.

## 1. Introduction

Nonalcoholic fatty liver disease (NAFLD) is one of the most prevalent chronic liver diseases, occurring in 25%-30% of the general population [1, 2]. NAFLD is defined as a clinicopathological syndrome, ranging from simple nonalcoholic fatty liver to nonalcoholic steatohepatitis (NASH) [3, 4]. As an inflection point for the deterioration of NAFLD, NASH can lead to the development of liver cirrhosis, liver failure, and hepatocellular carcinoma, which has emerged as the main cause of liver-related mortality and liver transplantation. NASH is characterized by hepatic steatosis, inflammation, hepatocytes ballooning, and variable degrees of fibrosis [5]. However, its molecular mechanisms are still not fully understood, and no effective control measures are available. Therefore, there is an urgent need to explore the pathophysiological mechanisms of NASH development.

Accumulating evidence has shown that excessive lipid accumulation-induced production of proinflammatory mediators and innate immune cell activation play the pivotal roles in the progression of NASH. Due to the secretion of inflammatory mediators such as chemokines, the innate immune cell macrophages and neutrophils are recruited into the liver and activated by damage-associated molecular patterns (DAMPs) released from injured hepatocytes, leading to aggravation of hepatic steatosis and fibrosis [6, 7]. As an important innate immune pattern recognition receptor (PRR), toll-like receptor 4 (TLR4) has been found to be upregulated in both NAFLD patients and animal models and is counted for the progression of hepatic steatosis, inflammation, and fibrosis [8–10]. It is well known that nuclear factor kappa B (NF-*κ*B), a key downstream molecule of TLR4 signal pathway, plays an important role for the transformation from simple steatosis to steatohepatitis [11, 12]. In the canonical pathway, NF-*κ*B proteins are bound and inhibited by I*κ*B proteins. DAMPs, metabolites such as free fat acid, and LPS trigger TLR4 signal to phosphorylate IL-1 receptor-associated kinases (IRAKs). Sequentially, the IKK*β* protein is activated, which phosphorylates I*κ*B protein, resulting in I*κ*B ubiquitination and degradation to release NF-*κ*B proteins. The freeing NF-*κ*B proteins such as p65 are activated by phosphorylation modification and translocated to the nucleus in which, as the key transcription factors, they induce these target genes expression such as tumor necrosis factor-*α* (TNF-*α*), interleukin-1*β* (IL-1*β*), and IL-6 [13, 14]. Therefore, targeting TLR4/NF-*κ*B signaling may be the underlying mechanisms and key therapeutic strategies of NASH development.

Milk fat globule-epidermal growth factor-factor 8 (MFG-E8), a secreted glycoprotein with two EGF-like domains, contains an RGD motif that is able to bridge phosphatidylserine on apoptotic cells and integrin *α*v*β*3 or *α*v*β*5 in phagocytes to accelerate phagocytosis of apoptotic cells, resulting in the inhibition of inflammatory responses [15–17]. It has been shown that MFG-E8 protects against liver fibrosis in mice by interfering with the action of transforming growth factor-*β*1 (TGF-*β*1) [18]. A recent study has found that MFG-E8 is highly expressed in human hepatocellular carcinoma (HCC) tissues and positively regulates HCC progression, and anti-MFG-E8 antibodies could effectively inhibit HCC progression and metastasis [19]. In addition, it has been demonstrated that serum MFG-E8 can be feasibly served as a diagnostic, and prognostic biomarker for HCC and hepatic MFG-E8 prevents from the development of hepatic steatosis and inflammation [20, 21]. Furthermore, it has been reported that MFG-E8 could attenuate the release of proinflammatory cytokines from immune cells by inhibiting TLR4 and NF-*κ*B pathways [22, 23] and is a key regulator of neutrophil infiltration in acute lung injury [24, 25]. However, the potential roles and mechanisms of MFG-E8 in the pathogenesis of NASH need to further be elucidated. Therefore, in the present study, we investigated the impact of MFG-E8 deficiency on the development of MCD-induced NASH model in mice and explored its potential mechanisms.

## 2. Materials and Methods

### 2.1. Chemicals and Reagents

The kits for alanine aminotransferase (ALT), aspartate aminotransferase (AST), and triglyceride (TG) were supplied by Nanjing Jiancheng Bioengineering Institute (Nanjing, China). Enzyme-linked immunosorbent assay (ELISA) kits for TNF-*α* and IL-1*β* were purchased from Bender MedSystems (Vienna, Austria). PE rat anti-mouse Ly6G was obtained from BD Biosciences (New Jersey, USA). Neutrophils and F4/80 antibodies were obtained from Thermo Scientific (Rockford, IL, USA). Rabbit anti-mouse TLR4 antibody, rabbit anti-mouse phospho-IRAK1, phospho-p38, phosphor-IKK*β*, IKK*β*, phospho-I*κ*B*α*, I*κ*B*α*, phospho-p65, p38, p65, *β*-actin, Lamin B1, and GAPDH were purchased from Abcam (Cambridge, UK). PE rat anti-mouse F4/80, FITC rat anti-mouse CD11b, and FITC rat anti-mouse CD45 antibodies were from Biolegend, Inc. (San Diego, USA). TLR4-specific antagonist TAK-242 was from MedChemExpress LLC (Shanghai, China). Palmitic acid (PA) was from Sigma-Aldrich (St. Louis, USA).

### 2.2. Animals and Animal Experiments

Male C57BL/6J (WT) mice (6–8 weeks, 20-25 g) were supplied by the Experimental Animal Center of Chongqing Medical University (Chongqing, China). MFG-E8 knockout (KO) mice were donated by Professor Tianpen Cui at Wuhan No. 1 Hospital affiliated to Tongji Medical College of Huazhong University of Science and Technology. The experimental animals were maintained in a specific standard laboratory condition (20-25°C, 55 ± 5% humidity and a circle of 12 h light/dark) and were fed regularly and watered ad libitum. All mice were acclimatized for at least 1 week prior to use. The experiments involving mice were performed in accordance with the guidelines of the Animal Care and Use Committee of Chongqing Medical University.

Mice were randomly divided into four groups (*n* = 6 in each group): CD-WT group, CD-KO group, MCD-WT group, and MCD-KO group. Mice in both MCD-WT and MCD-KO groups were fed MCD diet (purchased by Trophic Animal Feed High-tech Co., Ltd. Jiangsu, China) for 5 weeks to induce NASH. During the same period, mice in the other two groups were fed standard chow diet (CD) (purchased from Trophic Animal Feed High-tech Co., Ltd. Jiangsu, China). At the end of 5 weeks, all mice were sacrificed under anesthesia via sevoflurane, blood samples were collected from the retroorbital sinus, and liver tissues were collected for next analysis.

### 2.3. Cell Culture and Treatment

Liver tissues were perfused with Hank's balanced salt solution (HBS), followed by Dulbecco's modified Eagle's medium (DMEM) with 0.05% IV collagenases at 37°C. Primary murine hepatocytes were collected by centrifugation at 50 g for 2 min, then were seeded in coated collagen type I cultural plates with DMEM supplemented with 10% fetal bovine serum (FBS).

The primary murine hepatocytes from wild type or MFG-E8 knockout mice were stimulated with palmitic acid (PA) (0.5 mM) with or without TLR4 specific antagonist TAK-242 (10 nM) for 24 h. The supernatant was collected for cytokines assay; the cells were fixed by 75% ethanol and stained with Oil Red O solution. In other experiment, the cells were lysed with chloroform-methanol solution for intracellular TG measurement.

### 2.4. Biochemical Analysis

Blood samples were collected from mice and centrifuged to obtain serum. Liver samples were homogenized using a tissue homogenizer, and the supernatant was obtained by centrifugation. The activities of ALT and AST in serum and triglyceride (TG) in liver were measured using commercial assay kits according to the manufacturer's instructions.

### 2.5. Histological Analysis

Liver tissues were fixed in 4% paraformaldehyde, embedded in paraffin, and sliced at 5 *μ*m thickness. Subsequently, tissue sections were stained with hematoxylin and eosin (HE) staining, Masson's Trichrome staining, or Sirius Red staining and evaluated using light microscopy (Olympus, Tokyo, Japan). Steatosis, inflammation, and hepatocyte ballooning in HE staining of the liver were scored according to the NAS (NAFLD activity score).

### 2.6. Oil Red O Staining

Liver tissues were embedded in optimum cutting temperature (OCT) compound, sectioned at 15 *μ*m thickness, and fixed in 75% ethanol. Then, frozen sections were stained with Oil Red O solution and counterstained nuclear with hematoxylin.

### 2.7. Immunofluorescence of Macrophages and Neutrophils

Macrophages and neutrophils in the liver were visualized by immunofluorescence. Briefly, frozen sections (8 *μ*m) were incubated with primary antibody FITC rat anti-mouse F4/80 or PE rat anti-mouse Ly6G at 37°C in the dark for 1 h. Then, actin filaments were then labeled with ActinRed 555 or ActinGreen 488 (Thermo scientific, Rockford, USA) at 37°C for 1 h. Finally, sections were counterstained nuclear with 4,6-diamino-2-phenyl indole (DAPI) and analyzed by fluorescence microscopy (Olympus, Tokyo, Japan).

### 2.8. Flow Cytometry

Liver samples were grounded and digested with 0.05% IV collagenases at 37°C which were filtered and centrifuged at 50 g for 5 min for the supernatant. Then, the precipitate was obtained by centrifuging the supernatant at 500 g for 5 min and was resuspended by phosphate buffer. Next, liver nonparenchymal cells (NPC) in the precipitates were incubated with labeled CD45, F4/80, Ly6G and CD11b antibodies in the condition of 4°C and darkness. The infiltration of macrophages (CD11b^+^F4/80^+^) and neutrophils (CD45^+^Ly6G^+^) was detected by flow cytometry.

### 2.9. Enzyme-Linked Immunosorbent Assay (ELISA)

The levels of TNF-*α* and IL-1*β* in the liver and supernatants were measured by the ELISA kits following the manufacturer's protocols.

### 2.10. Reverse Transcription-Quantitative Polymerase Chain Reaction (RT-qPCR)

Briefly, 100 mg of liver tissue and 1 mL Trizol reagent (Invitrogen, CA, USA) were homogenized using a homogenizer. The lysed liver sample was incubated for 5 min to permit the nucleoprotein dissociation and added 200 *μ*L chloroform to mix, then securely cap the tube to incubate for 2 min. The sample was centrifuged at 4°C, 12000 g for 15 min, and the mixing contents were transferred 600 *μ*L of the colorless, upper aqueous phase containing the RNA to a new RNase-free tube. The RNA mixture was added an equal volume of 70% ethanol to vortex. After that, the supernatant was carefully discarded, and the precipitation was dried at room temperature, pipetting 200 *μ*L of RNase-free water to dissolve the pellet, and the total RNA solution was prepared after mixing well.

The complementary DNA (cDNA) was synthesized by the PrimeScript RT kit (Takara, Dalian, China). Quantitative real-time PCR was performed using the SYBR Green real-time PCR amplification kit (Takara, Dalian, China) following the manufacturer's protocol. The relative expression levels of all mRNAs were normalized to GAPDH expression. The primer sequences were listed as Table 1.

### 2.11. Western Blotting

The whole cell lysate, cytoplasmic, and nuclear soluble proteins from mouse liver tissues were separated by the RIPA lysis and Subcellular Protein Fractionation Kits (Thermo Fisher Scientific, Waltham, USA) according to the instructions. In brief, precisely weighed 100 mg of liver and 1000 *μ*L of newly prepared RIPA lysis buffer or cytoplasmic extraction buffer (CEB) were placed into prechilled tube on ice, homogenizing fully on ice. The supernatants (whole cell lysate or cytoplasmic extract) were transferred into clean prechilled tube to use. The pellet in CEB was added 225 *μ*L nuclear extraction buffer (NEB) containing protease inhibitors to vortex for 15 sec and incubate at 4°C for 30 min with gentle mixing. Then, the tube was centrifuged at 4°C, 5000 g for 5 min, and the supernatant (soluble nuclear extract) was collected. The protein concentrations were detected using the BCA assay kit.

Subsequently, proteins were subjected to electrophoresis through polyacrylamide-sodium dodecyl sulfate (SDS-PAGE) gel and transferred to polyvinylidene fluoride (PVDF) membrane. The membranes were then blocked with 5% bovine serum albumin (BSA) solution at room temperature for 1 h. Afterward, the membranes were incubated overnight at 4°C with appropriately diluted primary antibodies, followed by incubation with horseradish peroxidase- (HRP-) conjugated secondary antibodies for 1 h at room temperature. Eventually, antibody binding was displayed using an ECL chemiluminescent system and analyzed by Image Lab software.

### 2.12. Statistical Analysis

All data in this study were expressed as mean ± standard deviation (SD). Student's *t*-test was used to compare the difference between the two groups. One-way analysis of variance (ANOVA) followed by the Tukey *post hoc* test was used for multiple comparisons. *P* value < 0.05 was considered statistically significant.

## 3. Results

### 3.1. MFG-E8 Knockout Increased Serum ALT and AST Activities in NASH Mice

**Figure 1:**
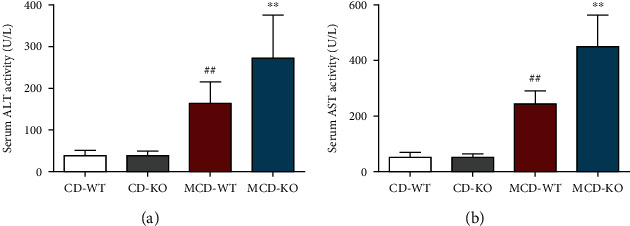
Effect of MFG-E8 knockout on serum ALT and AST activities in mice. Serum ALT (a) and AST (b) activities were measured after 5 weeks of feeding MCD diet or standard chew diet (CD) in wildtype (WT) or MFG-E8 knockout (KO). Data were expressed as mean ± SD, *n* = 6, ^##^*P* < 0.01, compared with CD-WT group; ^∗∗^*P* < 0.01, compared with MCD-WT group.

Serum ALT and AST activities are the crucial biochemical indicators for the evaluation of liver function. As shown in Figure 1, serum ALT and AST activities in the MCD-WT group were significantly higher than those in the CD-WT group (*P* < 0.01). Compared with the MCD-WT group, MFG-E8 knockout markedly increased serum ALT and AST activities (*P* < 0.01). In addition, there was no significant difference between the CD-WT and CD-KO groups, indicating that MFG-E8 knockout did not affect the liver function in mice.

### 3.2. MFG-E8 Knockout Aggravated MCD-Induced Hepatic Damage in Mice

**Figure 2:**
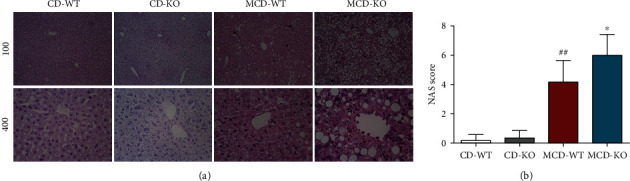
Effect of MFG-E8 knockout on liver injury induced by MCD in mice. (a) The liver pathological changes were determined by HE staining and observed under a microscope. (b) The NAS scores were analyzed. Arrow indicated inflammatory foci, arrowhead indicated hepatocyte ballooning, and asterisk indicated hepatocyte steatosis. Data were expressed as mean ± SD, *n* = 6, ^##^*P* < 0.01 compared with CD-WT group; ^∗^*P* < 0.05 compared with MCD-WT group.

To further confirm the effect of MFG-E8 on NASH, the histopathological changes of liver tissues were evaluated by HE staining and NAS scoring. As shown in Figure 2(a), there were not obvious pathological changes in both the CD-WT and CD-KO groups. In contrast, the apparent and diffuse hepatic steatosis with lobular inflammatory foci, as well as some ballooned hepatocytes were observed in the liver of the MCD-WT group, which were further aggravated in MFG-E8 knockout mice fed with MCD. Likewise, NAS scores showed that MFG-E8 knockout mice developed more severe hepatic pathological damage than wildtype mice after MCD diet for 5 weeks (Figure 2(b)).

### 3.3. MFG-E8 Knockout Deteriorated Hepatic Steatosis in NASH Mice

**Figure 3:**
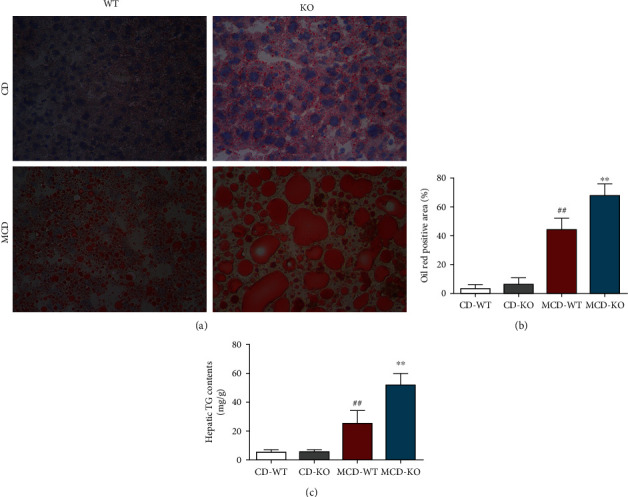
Effect of MFG-E8 knockout on MCD-induced hepatic lipid accumulation in mice. (a) Lipid accumulation in frozen sections of liver tissue was determined by Oil Red O staining (200x). (b) The Oil Red O staining positive area was measured and quantified. (c) The content of TG in the liver was assayed. Data were expressed as mean ± SD, *n* = 6, ^##^*P* < 0.01 compared with CD-WT group; ^∗∗^*P* < 0.01, compared with MCD-WT group.

To assess the effect of MFG-E8 on lipid droplet formation, Oil Red O staining and TG content measurement were performed. MCD diet induced a marked lipid deposition and fat vacuole accumulation in hepatocytes, which are typical histological features of steatosis. However, MFG-E8 knockout significantly deteriorated the size and number of hepatic lipid droplets (Figure 3(a)). Meanwhile, as shown in Figure 3(b), the Oil Red O staining positive area was significantly higher in the liver of MFG-E8 knockout mice compared to wildtype group (*P* < 0.01). Similarly, MFG-E8 knockout mice showed remarkably higher hepatic TG contents than control mice in MCD diet (*P* < 0.01) (Figure 3(c)).

### 3.4. MFG-E8 Knockout Exacerbated MCD-Induced Liver Fibrosis in Mice

**Figure 4:**
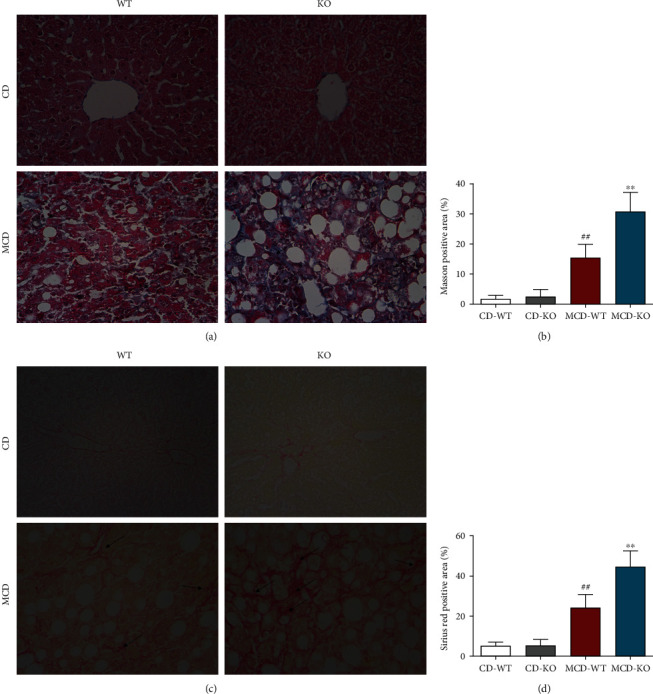
Effect of MFG-E8 knockout on MCD-induced liver fibrosis in mice. The liver section was performed by Masson's Trichrome staining and Sirius Red staining to assess the degree of liver fibrosis. (a) Images of Masson's Trichrome staining (200x). (b) The positive area of Masson's Trichrome staining. (c) Images of Sirius Red staining (200x). (d) The positive area of Sirius Red staining. Arrow indicated hepatic fibrosis changes. Data were expressed as mean ± SD, *n* = 6, ^##^*P* < 0.01 compared with CD-WT group; ^∗∗^*P* < 0.01, compared with MCD-WT group.

NASH is closely associated with liver fibrosis progressive. Thus, the extent of liver fibrosis was determined by Masson's Trichrome staining and Sirius Red staining. Compared with chow diet mice (CD-WT), MCD diet mice (MCD-WT) showed more significant liver fibrosis, which was drastically exacerbated by MFG-E8 knockout, as demonstrated by Masson's Trichrome stain (Figures 4(a) and 4(b), blue indicates collagen fibers) and Sirius Red stain (Figures 4(c) and 4(d), red indicates collagen fibers).

### 3.5. MFG-E8 Knockout Promoted Infiltration of Hepatic Macrophages and Neutrophils in NASH Mice

**Figure 5:**
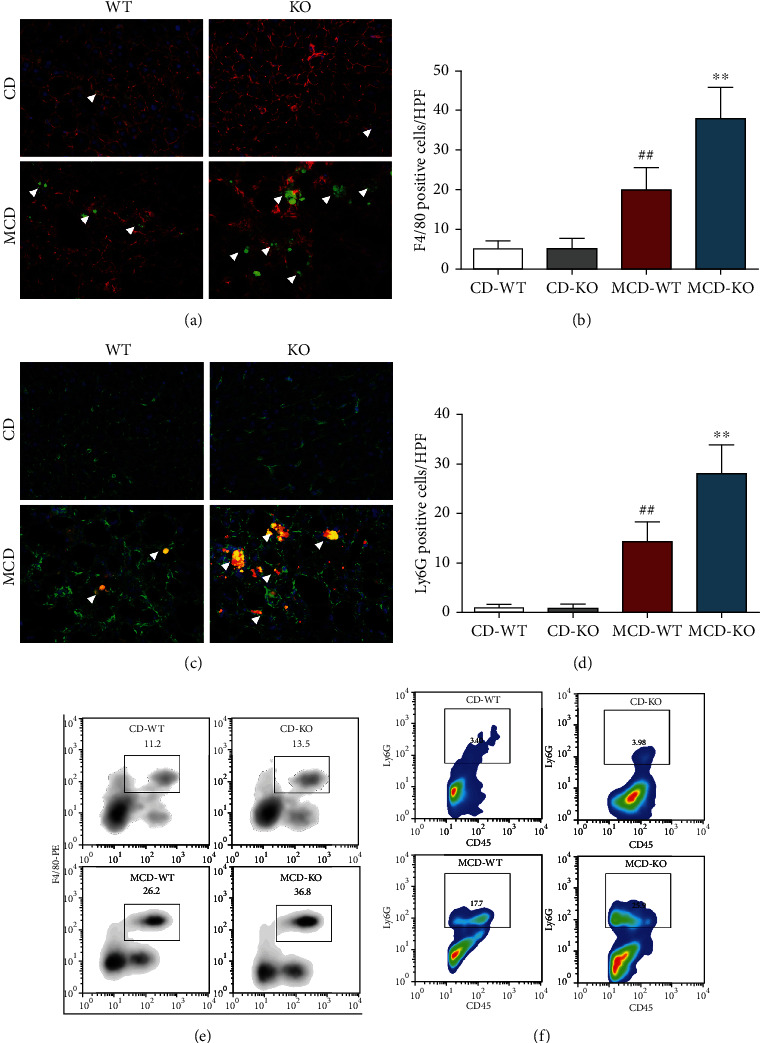
Effect of MFG-E8 knockout on hepatic macrophage and neutrophil infiltration in NASH mice. Hepatic macrophages and neutrophils were determined by immunofluorescence and flow cytometry using specific macrophage marker antibody F4/80 and neutrophil marker antibody Ly6G. (a) Representative immunofluorescence images of hepatic macrophages (200x), F4/80 positive cells were labeled with Green; F-actin was labeled with ActinRed 555. (b) F4/80 positive cells in high power field (HPF) were quantified. (c) Representative immunofluorescence images of hepatic neutrophils (200x), Ly6G positive cells were labeled with Red; F-actin was labeled with ActinGreen 488. (d) Ly6G positive cells in HPF were quantified. White arrowhead in the images indicated macrophage or neutrophil. (e) The CD11b^+^F4/80^+^ cells indicated as macrophages in representative flow cytometry analysis of hepatic nonparenchymal cells. (f) The CD45^+^Ly6G^+^ cells indicated as neutrophils in representative flow cytometry analysis of hepatic nonparenchymal cells. Data were expressed as mean ± SD, *n* = 6, ^##^*P* < 0.01 compared with CD-WT group; ^∗∗^*P* < 0.01, compared with MCD-WT group.

The infiltration of macrophages and neutrophils into the liver is one of the most crucial events in NASH development. Immunofluorescence staining analysis showed that compared to the MCD-WT group, MFG-E8 knockout mice exhibited augmented infiltration of F4/80^+^ macrophages and Ly6G^+^ neutrophils into the liver (Figures 5(a)–5(d)). Furthermore, as expected, flow cytometry analysis experiments showed a similar result that hepatic inflammatory cell (CD11b^+^F4/80^+^ macrophages and CD45^+^Ly6G^+^ neutrophils) numbers were markedly elevated in MCD diet-fed MFG-E8 knockout mice compared with MCD-WT group (Figures 5(e) and 5(f)).

### 3.6. MFG-E8 Knockout Enhanced the Production of Inflammatory Mediators in the Liver of NASH Mice

**Figure 6:**
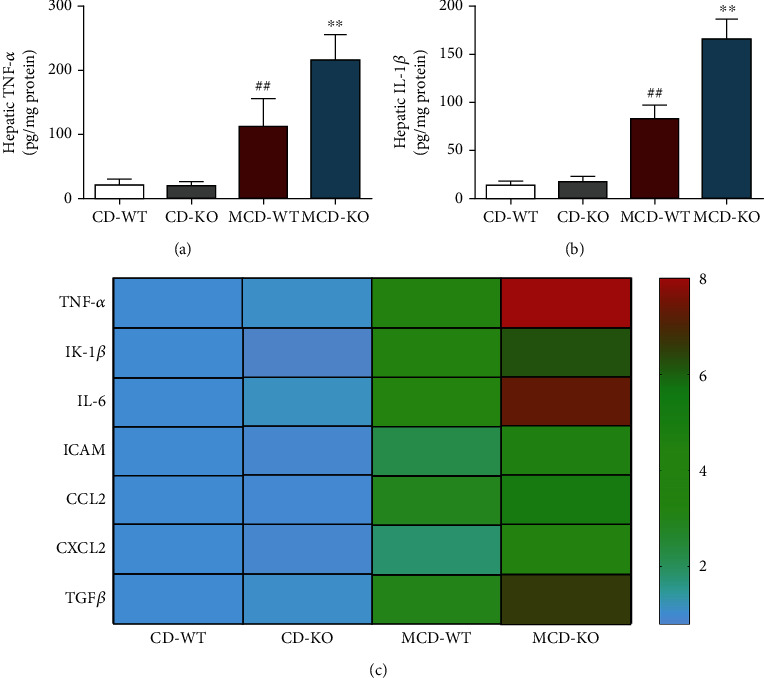
Effect of MFG-E8 knockout on hepatic inflammatory mediators in NASH mice. (a) TNF-*α* and (b) IL-1*β* protein levels in the liver were detected by ELISA. (c) The mRNA levels of hepatic TNF-*α*, IL-1*β*, IL-6, ICAM, CCL2, CXCL2, and TGF-*β* were measured by RT-qPCR. Data were expressed as mean ± SD, *n* = 6, ^##^*P* < 0.01 compared with CD-WT group; ^∗∗^*P* < 0.01, compared with MCD-WT group.

Further, the expression of proinflammatory mediators in the liver of NASH mice was analyzed by ELISA and RT-qPCR. As shown in Figures 6(a) and 6(b), after MCD diet for 5 weeks, the protein levels of hepatic TNF-*α* and IL-1*β* in MFG-E8 knockout mice were higher than that in wildtype mice (*P*<0.01). Consistently, the mRNA levels of inflammatory mediators TNF-*α* and IL-1*β*, as well as IL-6, ICAM, CCL2, CXCL2, and TGF-*β*, which were indicated as progressive inflammatory response and liver fibrosis, were significantly elevated in the liver of MFG-E8 knockout mice compared with the MCD-WT group (Figure 6(c)).

### 3.7. MFG-E8 Knockout Facilitated MCD-Induced Activation of TLR4/NF-*κ*B Signaling in the Liver of Mice

**Figure 7:**
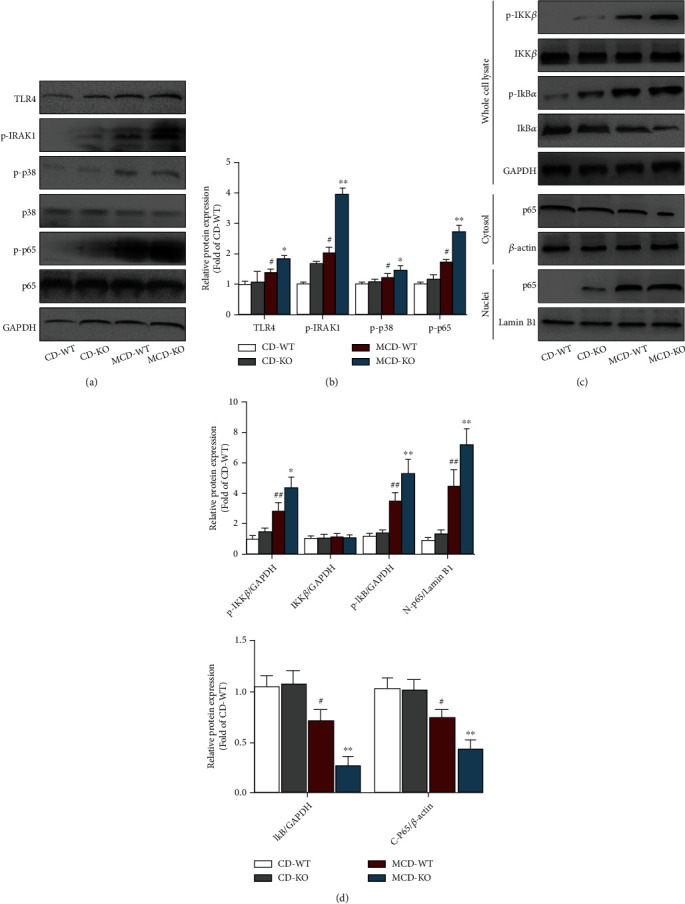
Effect of MFG-E8 knockout on TLR4/NF-*κ*B signaling pathway in the liver of NASH mice. The indicated proteins in whole cell lysates, cytoplasmic extraction, and nuclear extraction from the liver tissues were detected by Western blotting, respectively. Representative Western blotting (a) and quantification (b) of TLR4, p-IRAK1, p-p38, p38, p-p65, p65, and GAPDH protein levels in the liver. Representative Western blotting (c) and quantification (d) of p-IKK*β*, IKK*β*, p-I*κ*B*α*, I*κ*B*α*, and GAPDH in the whole cell lysates, p65, and *β*-actin in the cytoplasmic extraction, as well as p65 and Lamin B1 in the nuclear extraction from liver tissues. Data were expressed as mean ± SD, *n* = 3, ^#^*P* < 0.05, ^##^*P* < 0.01 compared with CD-WT group, ^∗^*P* < 0.05, ^∗∗^*P* < 0.01, compared with MCD-WT group.

To further explore the potential mechanism by which MFG-E8 knockout aggravated NASH progression, Western blotting was used to detect the activation of TLR4/NF-*κ*B signaling pathway. The results showed that the levels of TLR4, p-IRAK1, p-p38, and p-p65 proteins in the liver of the MCD-WT group were significantly higher than those of CD-WT group, but the total p38 and p65 protein levels were not significant. However, MFG-E8 knockout obviously upregulated the levels of hepatic TLR4, p-IRAK1, p-p38, and p-p65, indicating that MFG-E8 knockout enhanced MCD-induced TLR4 signal activation (Figures 7(a) and 7(b)). Accordingly, compared to MCD-WT group, MFG-E8 knockout significantly increased MCD-induced NF-*κ*B activation, as supported by enhancing the phosphorylation of IKK*β* and I*κ*B*α*, as well as increased I*κ*B*α* degradation. Moreover, the analysis of p65 in the subcellular localization indicated that freeing p65 was sharply translocated from cytoplasm into nucleus in the liver of MFG-E8 knockout mice compared with WT group fed by MCD (Figures 7(c) and 7(d)).

### 3.8. TLR4 Antagonist Rescued MFG-E8 Knockout-Enhanced TGs Synthesis and Proinflammatory Cytokine Production in Primary Hepatocytes Stimulated by PA

**Figure 8:**
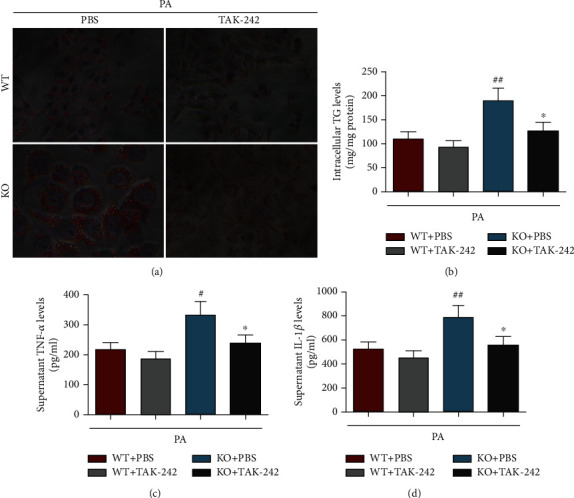
TLR4 antagonist rescued MFG-E8 knockout-enhanced TG synthesis and proinflammatory cytokine production in primary hepatocytes stimulated by palmitic acid (PA). The primary hepatocytes separated from WT or MFG-E8 knockout mice were stimulated by PA (0.5 mM) with or without TLR4 specific antagonist TAK-242 (10 nM) for 24 h, the lipid droplet was evaluated by Oil Red O staining (a), TG content was assayed by a commercial kit (b), and TNF-*α* (c) and IL-1*β* (d) protein levels in the supernatants were measured by ELISA. Data were expressed as mean ± SD, *n* = 6, ^#^*P* < 0.05, ^##^*P* < 0.01 compared with WT + TAK-242 group, ^∗^*P* < 0.05, compared with KO + PBS group.

To evaluate whether TLR4 mediated MFG-E8 knockout-aggravated NASH phenotype in MCD-fed mice, the primary hepatocytes separated from WT or MFG-E8 knockout mice were stimulated by PA with or without TLR4-specific antagonist TAK-242. In parallel with these results from *in vivo* animal experiment, MFG-E8 knockout hepatocytes showed higher lipid droplet formation and TG synthesis, as well as massive inflammatory cytokines TNF-*α* and IL-1*β* production compared with wildtype hepatocytes in response to PA stimulation. However, TLR4-specific antagonist TAK-242 significantly reverted MFG-E8 knockout-aggravated NASH phenotype, as indicated by decreased lipid droplet formation and TG synthesis, and weakened inflammatory cytokine production (Figure 8), suggesting that TLR4 might participate in the function of MFG-E8 on modulating NASH progression.

## 4. Discussion

NASH, a potentially progressive subtype of NAFLD that results in hepatocirrhosis and liver cancer, is closely associated with the metabolic syndrome and responsible for considerable economic burden globally [26, 27]. In the current study, we demonstrated that MFG-E8 plays an important role in the development of NASH. Our results found that MFG-E8 knockout significantly increased serum ALT and AST activities, exacerbated the histopathological liver injury as well as hepatic lipid accumulation, and promoted hepatic inflammatory responses and fibrosis in mice induced by MCD diet.

Liver fibrosis is a main histopathological feature of progressive NASH, exposing patients to a significant risk for cirrhosis and hepatocellular carcinoma. Previous several studies have suggested that MFG-E8 might be involved in the pathogenesis of fibrosis in various organs and tissues. For example, the expression of MFG-E8 was significantly downregulated in the sclerotic skin lesions in systemic sclerosis patients with skin fibrosis, in the smooth muscle cells surrounding the fibrotic respiratory tracts of asthma patients, and in the cirrhotic livers [18, 28, 29]. As indicated, MFG-E8 KO mice developed severe pulmonary fibrosis and skin fibrosis upon intratracheal bleomycin administration [28, 30]. Similarly, our results showed that MFG-E8 knockout mice exhibited more severe hepatic fibrosis compared to the MCD-WT group.

Mounting evidence has revealed that the imbalance lipid metabolism is the main etiology of hepatic steatosis and fibrosis. Excessive lipid accumulation in the liver induces metabolic stress and causes lipotoxicity, resulting in liver parenchymal cell death. The hepatocyte-death-released DAMPs activate innate immune signaling by PPRs, which trigger sustained inflammatory cascade and further worsen metabolic disorders and, finally, drive NASH progression. Thus, dissection of lipid metabolic disorder and excessive innate immune reaction is important for exploring the underlying mechanisms or identifying novel therapeutic targets of NASH development [31–33]. In this study, MFG-E8 knockout deteriorated hepatic steatosis in NASH mice, indicating that MFG-E8 may lower hepatic lipid production through a direct or indirect molecular mechanism. However, a previous study has showed that MFG-E8 could promote fatty acid uptake and cause obesity in mice by inducing the translocation of CD36 and FATP1 into cell surface. This data seems be controversial with our present results. However, this report showed that MFG-E8 mainly affects adipocytes but not hepatocytes in the absorption of fatty acid from blood [34]. In addition, in our experiment, the MCD diet but not high fatty diet- (HFD-) induced NASH model was used. The two NASH models have obvious different phenotype and pathogenesis. In the MCD model, lack of methionine and choline in diet interrupts the VLDL assembly, which leads to decreased TG secretion, resulting in hepatic lipid accumulation [35]. In fact, lipid metabolic disorder is involved in an imbalance between hepatic lipid input and output. Recently, Zhang et al. reported that MFG-E8 improved hepatic steatosis and inflammation through inhibiting apoptosis signal-regulating kinase 1 (ASK1) and mitogen-activated protein kinase (MAPK) signaling in hepatocytes [21]. Considering that ASK1 and MAPKs are downstream molecules of TLR4 signal pathway, and the lipid output but not its input is declined in MCD-induced NASH, we speculate that MFG-E8 does not directly regulate lipid metabolism but might block inflammatory cascade-worsened metabolic disorders by inhibiting innate immune TLR4 signal.

It is acknowledged that metabolic inflammation is tightly regulated by innate immune signal. Hepatic macrophages and neutrophils have been identified as the main innate immune cells in NAFLD [36, 37]. Infiltrating macrophages and neutrophils secrete proinflammatory cytokines and chemokines that promote the progression of liver inflammation and fibrosis and aggravate hepatic steatosis [38–40]. Established data suggested that MFG-E8 could inhibit the production of proinflammatory mediators and alleviate macrophage and neutrophil infiltration [23, 41–43]. Consistently, in the present study, by immunofluorescence staining and flow cytometry, we found that MFG-E8 knockout exhibited more severe hepatic macrophages and neutrophil infiltration in the liver of NASH mice. In addition, the RT-qPCR and ELISA analysis also indicated that MFG-E8 knockout upregulated the expression of inflammatory mediators.

TLR4 and NF-*κ*B play a critical role in the innate immune inflammatory responses and are closely related to the production of inflammatory mediators and cellular damage. It has been well demonstrated that activation of TLR4/NF-*κ*B signaling pathway aggravates inflammatory responses and promotes NASH progression [10, 44–46]. We previously reported that mice fed by an MCD diet exhibited severe inflammation and liver injury through upregulating the expression of proinflammatory cytokines and chemokines, which coincided with activation of TLR4/NF-*κ*B signaling pathway in the liver [14]. Additionally, inhibition of TLR4 or NF-*κ*B activation has been shown to exert the beneficial therapeutic role in several NASH mouse models [47, 48]. In other inflammatory models, MFG-E8 is also indicated to be effective for attenuating inflammatory response through inhibiting the activation of TLR4/NF-*κ*B pathway [22, 23]. Notably, several previous studies have shown that the expression of MFG-E8 is downregulated by activation of TLR signal *in vitro* and *in vivo*, indicating that there might be negative feedback mutual interaction between TLR signal and MFG-E8 [49, 50]. In the current study, overactivated TLR4/NF-*κ*B signaling pathway, as well as the elevated levels of inflammatory mediators, was observed in the MCD-KO group, suggesting that the effect of MFG-E8 in NASH might be mediated by TLR4/NF-*κ*B signal pathway. Furthermore, using a primary hepatocyte model, we found that specific inhibiting of TLR4 could effectively rescue MFG-E8 knockout-aggravated NASH phenotype. Thus, our data suggested that MFG-E8 knockout promoted hepatic steatosis, inflammation, and fibrosis in MCD-induced NASH, which might be by activation of TLR4/NF-*κ*B signaling pathway.

## 5. Conclusion

In conclusion, we confirmed that MFG-E8 knockout exacerbated the development of NASH, and the underlying mechanism may be related to the activation of TLR4/NF-*κ*B signaling pathway, which led to hepatic inflammatory cell infiltration and proinflammatory mediator production. Considering the role of MFG-E8 knockout in promoting liver inflammation and fibrosis, it is reasonable to expect that targeting MFG-E8 may be a promising strategy for improving NASH outcome.

## Figures and Tables

**Table 1:** The primers of RT-qPCR.

Target gene	Forward primer	Reverse primer
TNF-*α*	5′-CAGGCGGTGCCTATGTCTC-3′	5′-CGATCACCCCGAAGTTCAGTAG-3′
IL-1*β*	5′-GAAATGCCACCTTTTGACAGTG-3′	5′-TGGATGCTCTCATCAGGACAG-3′
IL-6	5′-CTGCAAGAGACTTCCATCCAG-3′	5′-AGTGGTATAGACAGGTCTGTTGG-3′
ICAM-1	5′-GTGATGCTCAGGTATCCATCCA-3′	5′-CACAGTTCTCAAAGCACAGCG-3′
CCL2	5′-TAAAAACCTGGATCGGAACCAAA-3′	5′-GCATTAGCTTCAGATTTACGGGT-3′
CXCL2	5′-CCAACCACCAGGCTACAGG-3′	5′-GCGTCACACTCAAGCTCTG-3′
TGF-*β*	5′-CCACCTGCAAGACCATCGAC-3′	5′-CTGGCGAGCCTTAGTTTGGAC-3′
GAPDH	5′-TGACCTCAACTACATGGTCTACA-3′	5′-CTTCCCATTCTCGGCCTTG-3′

## Data Availability

The datasets used and/or analyzed during the current study are available from the corresponding author on reasonable request.
